# Ferric derisomaltose augments intrinsic skeletal muscle electron transport chain activity in heart failure: A FERRIC‐HF II molecular substudy

**DOI:** 10.1002/ejhf.70028

**Published:** 2025-09-08

**Authors:** Mohamad F. Barakat, Nelson Amaral, Daniel Brayson, George Amin‐Youssef, Huda Abu‐Own, Salma Ayis, Francesco Papalia, Fadi Jouhra, Adam Nabeebaccus, Mark Monaghan, Gerry Carr‐White, Alison Sleigh, Geoffrey Charles‐Edwards, Ajay M. Shah, Graham J. Kemp, Andrew J. Murray, Darlington O. Okonko

**Affiliations:** ^1^ School of Cardiovascular & Metabolic Medicine and Science, James Black Centre, King's College London British Heart Foundation Centre of Excellence London UK; ^2^ School of Life Sciences, University of Westminster; ^3^ King's College Hospital NHS Foundation Trust London UK; ^4^ School of Population Health and Environmental Sciences, King's College London UK; ^5^ Guy's and St Thomas' NHS Foundation Trust London UK; ^6^ Wolfson Brain Imaging Centre, University of Cambridge School of Clinical Medicine Cambridge UK; ^7^ Wellcome Trust‐MRC Institute of Metabolic Science, University of Cambridge Cambridge UK; ^8^ School of Imaging Sciences and Biomedical Engineering, King's College London UK; ^9^ Medical Physics, Division of Imaging Sciences and Biomedical Engineering Guy's and St Thomas'‐NHS Foundation Trust London UK; ^10^ Liverpool Magnetic Resonance Imaging Centre and Institute of Life Course and Medical Sciences, University of Liverpool Liverpool UK; ^11^ Department of Physiology, Development and Neuroscience University of Cambridge Cambridge UK

**Keywords:** Heart failure, Iron, Muscle, Respirometry, Energetics, Mitochondria

## Abstract

**Aims:**

Skeletal muscle energetic augmentation might be a mechanism via which intravenous iron improves symptoms in heart failure, but no direct measurement of intrinsic mitochondrial function has been performed to support this notion. This molecular substudy of the FERRIC‐HF II trial tested the hypothesis that ferric derisomaltose (FDI) would improve electron transport chain activity, given its high dependence on iron–sulfur clusters which facilitate electron transfer during oxidative phosphorylation.

**Methods and results:**

Vastus lateralis skeletal muscle biopsies were taken before and 2 weeks after randomization. Mitochondrial complex I, II, and I&II respiration were quantified with respirometry of permeabilized fresh skeletal muscle biopsies. Net respiratory capacities, reflecting respiration that is truly available for adenosine triphosphate generation, were calculated by subtracting non‐phosphorylating LEAK respiration. Complex I–V and myoglobin protein levels, and skeletal muscle fibre type composition were assayed. Patients randomised to FDI (*n* = 21) or placebo (*n* = 19) were similar (age 66 ± 13 years, 73% men, left ventricular ejection fraction 37 ± 8%, 48% New York Heart Association class III, 50% diabetic). After 2 weeks, total complex I‐linked respiration (0.33 [interquartile range 0.24–0.37] vs. 0.19 [0.06–0.27] nmol/min/mg, *p* = 0.03) and net complex I‐linked respiration (0.21 [0.16–0.24] vs. 0.11 [0.04–0.16] nmol/min/mg, *p* = 0.01) were higher in patients allocated to FDI. There was no intergroup difference in other respiratory states, in mitochondrial abundance as reflected by complex I–V protein levels, and in skeletal muscle myoglobin and oxidative fibre type content.

**Conclusions:**

Iron repletion induces an early, selective, and potentially direct enhancement of mitochondrial complex I‐dependent respiration in the skeletal muscle of heart failure patients. This could be harnessed to optimize repletion protocols to maximize patient benefits.

## Introduction

Intravenous (IV) iron repletion can improve symptoms and exercise performance in chronic heart failure,^1^, ^2^ but the underlying mechanisms are incompletely understood. Deeper elucidation of how benefits accrue might facilitate the optimization of repletion protocols to maximize patient benefits. In the FERRIC‐HF II trial, ferric derisomaltose (FDI; previously known as iron isomaltoside) augmented skeletal muscle energetics, as evidenced by an 8 s faster phosphocreatine (PCr) recovery halftime on phosphorous magnetic resonance spectroscopy (^31^P MRS).^3^ Frise *et al*.^4^ reported similar magnitudes of treatment effect in otherwise healthy iron‐deficient individuals. However, because the kinetics of PCr recovery is also influenced by factors extrinsic to mitochondria, it is still unclear whether IV iron repletion enhances intrinsic mitochondrial electron transport chain (ETC) activity.

Electron flux through the ETC is the molecular lynchpin of oxidative phosphorylation which provides over 90% of skeletal muscle adenosine triphosphate (ATP).^5^ Substrate oxidation is the initial source of electrons which enter the ETC via complex I, complex II, or fatty acid specific pathways. Convergent electron flow to coenzyme Q is then linearly cascaded to molecular oxygen via complex III, cytochrome c and complex IV. As an exergonic process, this sequential electron transfer powers proton translocation across the inner mitochondrial membrane which contributes to the proton‐motive force that drives ATP synthesis. Iron sulphur (Fe‐S) cluster and haem prosthetic groups are implicated in electron transfer; for example, electrons are passed along 7 low to high potential Fe‐S clusters in complex I.^6^, ^7^ Consequently, iron deficiency, even in the absence of anaemia, is linked to suppressed energetics with impairments in varying ETC complexes reported in observational studies.^8^, ^9^, ^10^ Because complex I is the most rate‐limiting enzyme of the ETC and has the highest content of [4Fe‐4S] clusters that are more labile than [2Fe‐2S] clusters and haem,^6^, ^11^, ^12^ iron repletion might induce an early and selective enhancement of complex I‐linked respiration. Alternatively, IV iron might globally boost the ETC by restoring the lower skeletal muscle content of myoglobin (which contains haem) and oxidative fibres observed in some murine iron deficiency models.^9^, ^10^ To date, no study has directly quantified the effect of IV iron repletion on intrinsic ETC activity in humans.

Mitochondrial respirometry is the gold standard means of analysing specific ETC complex activities *in situ*.^13^ With the use of polarographic sensors, the rate of oxygen consumption due to tissue metabolism is recorded in real‐time. Saturating concentrations of substrates, uncouplers, and inhibitors can be used to interrogate multiple respiratory pathways to establish the functional capacity and efficiency of mitochondria. In the FERRIC‐HF II trial, respirometry was performed on permeabilized fresh skeletal muscle biopsies to test the hypothesis that FDI enhances intrinsic ETC activity independently of skeletal muscle myoglobin levels and fibre type composition.

## Methods

### Study design

This is a prespecified substudy of FERRIC‐HF II (EudraCT 2012–005592‐13) for which the design, methods, and primary results have been reported.^3^ In brief, the trial enrolled 40 patients with symptomatic chronic heart failure, an left ventricular ejection fraction ≤45%, and iron deficiency as defined by the FERRIC‐HF criteria of ferritin <100 μg/L or 100–300 μg/L with transferrin saturation (TSAT) <20%. Study participants were randomized to a double‐blinded total dose infusion of FDI (*n* = 21) or saline placebo (*n* = 19) with treatments masked with opaque IV infusion sets. Endpoints were assessed at baseline and at 2 weeks post‐treatment to minimize the influence of skeletal muscle exercise adaptation on results. Regulatory approval was given by the South‐Central Berkshire ethics committee, the UK Medicines and Healthcare products Regulatory Agency, and the local research governance board.

### Skeletal muscle biopsies

A percutaneous biopsy of the distal region of the right vastus lateralis (10 cm above the patella and ∼2 cm from the fascia) was obtained under local anaesthesia with 1% lignocaine using a Weil‐Blakesley conchotome. A portion of the biopsy was immediately placed in an ice‐cold preservation medium (BIOPS) for respirometry. This solution contained 10 mM Ca‐EGTA buffer, 0.1 μM free calcium, 20 mM imidazole, 20 mM taurine, 50 mM K‐MES, 0.5 mM DTT, 6.56 mM MgCl_2_, 5.77 mM ATP and 15 mM PCr (pH 7.1).^14^ The remaining biopsy was immediately snap frozen in liquid nitrogen and stored at −80°C for later analyses.

### Preparation of permeabilized myofibers

After 30–60 min on ice‐cold BIOPS, skeletal muscle fibre bundles were gently dissected free of all visible blood and connective tissue. Myofibre bundles were then placed in 2 ml of ice‐cold BIOPS and permeabilized by incubating with 50 μg/ml saponin with gentle agitation at 4°C for 20 min. After permeabilization, fibre bundles were washed thrice by transfer and agitation in 2 ml of ice‐cold MiR05, a mitochondrial respiration buffer containing 110 mM sucrose, 60 mM potassium lactobionate, 20 mM taurine, 20 mM HEPES, 10 mM KH_2_PO_4_, 3 mM MgCl_2_, 0.5 mM EGTA, and 1 g/L BSA (pH 7.1).^14^ Bundles were then blotted on filter paper and weighed on a precision micro‐balance before placement into a respirometry chamber (Hansatech Oxytherm+ system, Hansatech instruments, Norfolk, UK) containing 2 ml of MiR05.

### Respirometry

Duplicate mitochondrial respirometry runs were performed at 37°C using a substrate‐uncoupler‐inhibitor titration protocol.^14^ On the morning of each experiment, a background calibration of each polarographic (Clarke electrode) oxygen sensor was done to ensure instrument stability. After baseline (non‐mitochondrial) respiration was recorded in the absence of substrates, this injection protocol was followed: glutamate (2 mM) and malate (10 mM) to quantify LEAK (non‐phosphorylating) respiration supported by complex I, ADP (saturating concentration 5 mM) for complex I‐linked respiration, succinate (10 mM) for complex I&II‐linked respiration, rotenone (0.5 μM) for complex II‐linked respiration, cytochrome *c* (10 μM) to assess outer mitochondrial membrane integrity, and carbonyl cyanide‐p‐trifluoromethoxyphenylhydrazone (0.5 μM stepwise titrations) for maximal uncoupled electron transfer system capacity (ETS). Respiration was recorded for 5–10 min after injections with oxygen fluxes measured at steady state and corrected for skeletal muscle wet mass (nmol/min/mg). Baseline respiration was subtracted from each respiratory state. Net respiratory rates (complex I_NET_, complex I&II_NET_, ETS_NET_, complex II_NET_), reflecting ETC activity that is truly available for ATP generation,^15^ were calculated by subtracting LEAK respiration. Mitochondrial efficiency was assessed by calculating the respiratory control ratio for ADP (RCR_ADP_ = complex I/LEAK), and the phosphorylation system control ratio (P/E = complex I&II/ETS). Respirometry was performed by a single experienced blinded operator.

### Protein immunoblots

Snap frozen skeletal muscle samples were homogenized in a buffer containing 25 mM Tris–HCl, 150 mM NaCl, 2 mM EGTA, 5 mM EDTA, 0.5% NP‐40 and protease/phosphatase‐inhibitor cocktails (1:100 v/v; Sigma‐Aldrich). After quantification of total protein content with Bradford reagent 80, 5x Laemmli buffer was added to each sample which were then loaded onto 7.5%–15% SDS‐polyacrylamide gels, electrophoresed in a buffer containing 0.1% SDS v/v, transferred to a nitrocellulose membrane, and stained with Ponceau S. Membranes were incubated with a primary antibody cocktail that targeted complex I‐V (ab110411, Abcam), and a single monoclonal against myoglobin (ab77232, Abcam). This was followed by anti‐mouse or anti‐rabbit fluorochrome‐conjugated secondary antibodies (IRDye 680RD and 800RD, Li‐Cor). Images were acquired using the Odyssey® CLx imager (Li‐Cor). Image analysis and densitometric quantification of specific protein bands were performed using Image Studio 2.1 (Li‐Cor).

### Indirect immunofluorescence staining of myosin heavy chain isotypes

For skeletal muscle fibre typing, skeletal muscle biopsies were embedded in optimal cutting temperature compound and cryosectioned to 10 μm thickness and mounted onto high quality polylysine or superfrost slides. Sections were fixed by immersion in a Coplin jar in pre‐chilled 100% methanol at −20°C, permeabilized in 0.5% Nonident P‐40, washed, and then blocked with 3% donkey serum. Primary antibodies targeting slow skeletal myosin heavy chain (ab11083, Abcam) were applied and incubated overnight at 4°C. Specifically bound antibodies were detected with a secondary anti‐mouse fluorophore‐conjugated antibody (Li‐Cor) and DAPI was added to visualize DNA. Immunofluorescence was analysed using wide‐field fluorescence microscopy (IX81 microscope, Olympus) by an experienced microscopist at 20x eye‐lens magnification. Myofibres positive for slow myosin heavy chain (oxidative fibres) were counted.

### Statistics

Statistical analyses were prespecified and followed the intention‐to‐treat principle. Data are presented as mean ± standard deviation, median (25th–75th percentile), or frequencies (%). Normality was checked with the Shapiro–Wilk test. Pearson's or Spearman rank coefficients were determined to assess correlations. Intragroup comparisons were made with a paired Student's *t*‐test or a Wilcoxon signed‐rank test. Intergroup comparisons at 2 weeks were conducted with an analysis of covariance (ANCOVA) with baseline variable values as covariate. The treatment effect (change in FDI arm – change in placebo arm) was calculated. Missing data were imputed using the mean value for the treatment group. Sensitivity analyses without imputation and with a *post hoc* Markov chain‐Monte Carlo imputation with 10 iterations were performed and showed consistent results unless otherwise stated. All statistical tests were two‐sided and we judged a *p*‐value <0.05 significant. All analyses were carried out using SPSS version 27 (IBM SPSS, New York, NY, USA).

## Results

### Baseline skeletal muscle mitochondrial respiration

**Figure 1:**
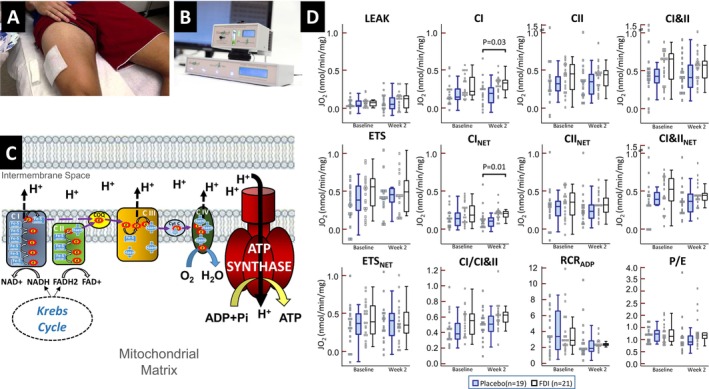
Skeletal muscle respiration. Fresh muscle bundles obtained from vastus lateralis biopsies (*A*) were subjected to respirometry on a Hansatech instrument (*B*) with the activity of the electron transport chain (*C*) compared between the ferric derisomaltose (FDI) and placebo groups (*D*). Substrate oxidation releases energy‐rich electrons to the reducing equivalents, nicotinamide adenine dinucleotide (NADH) and flavin adenine dinucleotide (FADH2). NADH receives electrons for complex I (CI)‐linked respiration while FADH2 receives electrons for complex II (CII)‐linked respiration. Iron–sulfur (Fe‐s) cluster and haem prosthetic groups in ETC proteins CI, CII, coenzyme Q (CoQ), complex III (CIII), and cytochrome C (Cyt C) facilitate electron transfer which releases redox energy that fuels the generation of the proton gradient that drives ATP synthesis. Box plots show the median (line), 25th and 75th percentiles (box), and the range (whiskers). ADP, adenosine diphosphate; CI&II, complex I&II; ETS, electron transport system capacity; FDI, ferric derisomaltose; JO_2_, oxygen consumption; RCR, respiratory control ratio; P/E, phosphorylation system control ratio.

The baseline characteristics of the 40 patients were broadly similar in the treatment arms (*Table* *1*). Of the 40 randomized patients, one in the FDI arm did not attend a week 2 visit because of hospitalization, and four in the FDI arm and two in the placebo arm declined a week 2 skeletal muscle biopsy. Baseline values for all respirometry indices in the total population are summarized in *Table* *2*. These were broadly similar between the treatment groups (*Figure* *1*).

**Table 1:** Baseline characteristics

	Placebo (*n = 19*)	Ferric derisomaltose (*n = 21*)
Age, years	62 ± 13	70 ± 12
Men	13 (68)	16 (76)
Caucasian ethnicity	14 (74)	17 (81)
Body mass index, kg/m^2^	30 ± 7	29 ± 4
Ischaemic aetiology	10 (53)	11 (52)
Hypertension	13 (64)	13 (62)
Diabetes mellitus	10 (53)	10 (48)
Atrial fibrillation/flutter	4 (21)	6 (29)
Left ventricular ejection fraction, %	37 ± 8	37 ± 8
NYHA class III	10 (53)	9 (43)
Systolic blood pressure, mmHg	122 ± 17	124 ± 16
Diastolic blood pressure, mmHg	71 ± 14	73 ± 10
Heart rate, bpm	71 ± 11	72 ± 10
6‐min walking distance, m	313 ± 67	324 ± 79
Resting skeletal muscle pH	7.01 ± 0.04	7.02 ± 0.03
PCr recovery halftime, s	33 ± 9	35 ± 12
Ferritin, ng/ml	59 (39–79)	34 (18–50)
<30	4 (21)	6 (29)
<100	19 (100)	21 (100)
Serum iron, μmol/L	11 ± 6	13 ± 4
≤13	14 (74)	11 (52)
Transferrin saturation, %	18 ± 10	21 ± 8
<20	13 (68)	10 (48)
Soluble transferrin receptor, mg/L	4.0 ± 1.5	3.6 ± 0.8
Haemoglobin, g/L	128 ± 20	130 ± 15
Creatinine, μmol/L	108 ± 34	121 ± 39
C‐reactive protein, mg/L	5 (1–10)	6 (3–9)
Medications		
Diuretics	12 (63)	14 (67)
ACE‐inhibitor, ARB, or ARNI	17 (89)	16 (76)
Beta‐blockers	16 (84)	18 (86)
Mineralocorticoid receptor antagonists	12 (63)	12 (57)
Digoxin	4 (21)	6 (29)
Anticoagulants	3 (16)	6 (29)
Antiplatelets	13 (68)	13 (62)

Data are mean ± standard deviation, n (%), or median (25th–75th percentile).

ACE, angiotensin‐converting enzyme; ARB, angiotensin receptor blocker; ARNI, angiotensin receptor–neprilysin inhibitor; NYHA, New York Heart Association; PCr, phosphocreatine.

**Table 2:** Baseline skeletal muscle respiration

Variable	
LEAK	0.06 ± 0.06
Complex I	0.20 (0.13–0.36)
Complex II	0.36 (0.23–0.54)
Complex I&II	0.52 ± 0.29
ETS	0.45 ± 0.27
Complex I_NET_	0.15 (0.08–0.29)
Complex II_NET_	0.31 ± 0.22
Complex I&II_NET_	0.45 ± 0.26
ETS_NET_	0.39 ± 0.25
RCR_ADP_	2.98 (2.08–5.80)
PE	1.18 ± 0.38
Complex I/Complex I&II	0.46 (0.34–0.64)

Variables are expressed in nmol/min/mg except for RCR_ADP_, PE, and complex I/complex I&II which are dimensionless ratios. Data are mean ± standard deviation or median (25th–75th percentile).

ADP, adenosine diphosphate; ETS, electron transport system capacity; PE, phosphorylation system control ratio; RCR, respiratory control ratio.

### Ferric derisomaltose and skeletal muscle mitochondrial respiration

From baseline to week 2, complex I_NET_ activity, which reflects respiration above non‐phosphorylating LEAK respiration that generates ATP with electron provision via complex I, increased by 12% (0.02 nmol/min/mg) in the FDI group and decreased by 22% (0.03 nmol/min/mg) in the placebo arm. The primary comparison of complex I_NET_ respiration at 2 weeks was higher in patients randomized to FDI with a treatment effect of 0.05 nmol/min/mg (*p* = 0.01). This result persisted after *post hoc* adjustment for both baseline complex I_NET_ and week 2 systolic blood pressure (*p* = 0.01) or week 2 resting skeletal muscle pH (*p* = 0.01), and in the absence of imputation (treatment effect 0.09 nmol/min/mg, *p* = 0.03). Individual patient values at baseline and week 2 for each group are shown in online supplementary *Figure* *S1*. No significant correlations were observed between changes in total and net complex I activities with baseline iron indices (TSAT, ferritin, serum iron, soluble transferrin receptor) or the deviation of haemoglobin from World Health Organization criteria (online supplementary *Figure* *S1*).

Similarly, from baseline to week 2, total complex I‐linked respiration increased by 51% (0.11 nmol/min/mg) in the FDI group but also increased by 27% (0.04 nmol/min/mg) in the placebo arm. At 2 weeks, complex I‐linked respiration was higher in patients randomized to FDI with a treatment effect of 0.07 nmol/min/mg (*p* = 0.02). This result persisted after *post hoc* adjustment for both baseline complex I‐linked respiration and week 2 systolic blood pressure (*p* = 0.02) or week 2 resting skeletal muscle pH (*p* = 0.02) but not in the absence of imputation (treatment effect 0.01 nmol/min/mg, *p* = 0.12). FDI had no impact on other indices of mitochondrial respiration and efficiency although there was a trend for increased P/E (*p* = 0.06) and complex I/complex I&II ratio (*p* = 0.10) with FDI.

### Ferric derisomaltose and skeletal muscle complex I‐IV protein levels

**Figure 2:**
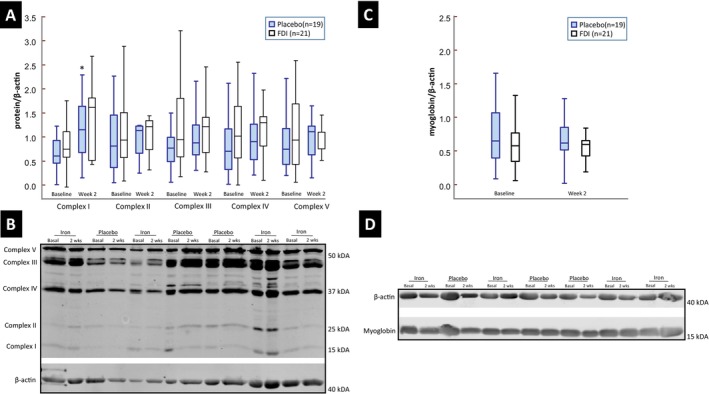
Levels of mitochondrial complex proteins and myoglobin. Complex I to V protein levels normalized to β‐actin are shown at baseline and at week 2 for the ferric derisomaltose (FDI) and placebo groups (*A*), with representative western blots (*B*). Similarly, myoglobin protein levels normalized to β‐actin are also shown at baseline and at week 2 for the randomized treatment groups (*C*) with representative western blots (*D*). *p = 0.008 against baseline. Box plots show the median (line), 25th and 75th percentiles (box), and the range (whiskers).

Expression of mitochondrial complex I‐V protein levels not only determine the activity of these enzymes but are also surrogates of mitochondrial abundance. From baseline to week 2, skeletal muscle complex I expression increased by 126% (*p* = 0.15) in the FDI group and by 98% (*p* = 0.008) in placebo patients with no intergroup difference at week 2 (*Figure* *2A,B*). There were no differences in complex II‐V expression.

### Ferric derisomaltose and skeletal muscle myoglobin and oxidative fibre type content

**Figure 3:**
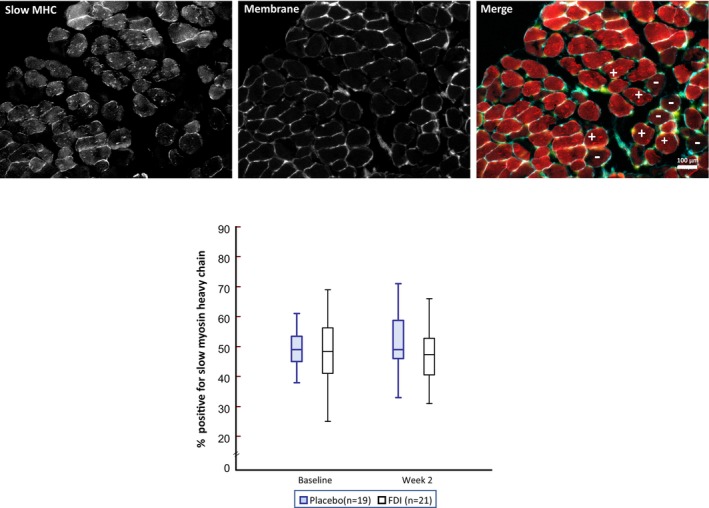
Skeletal muscle fibre type composition. The percentage of skeletal muscle fibres expressing slow myosin heavy chain (MHC) isoforms are shown at baseline and at 2 weeks for the ferric derisomaltose (FDI) and placebo groups. Representative images of immunostained skeletal muscles are shown above with fibres positive (+) and negative (−) for slow MHC isoforms (= oxidative fibres) marked in the merged image. Scale bar: 100 μm. Box plots show the median (line), 25th and 75th percentiles (box), and the range (whiskers).

Skeletal muscle myoglobin levels (*Figure* *2C,D*) and the proportion of slow twitch oxidative muscle fibres were not different between the treatment groups at week 2 (*Figure* *3*).

## Discussion

Skeletal muscle energetic augmentation might be a mechanism via which IV iron confers benefits in heart failure, but this notion has yet to be substantiated by direct measurement of intrinsic mitochondrial function, and the specific bioenergetic pathway(s) involved are unclear. To the best of our knowledge, this is the first study to show that iron repletion with FDI induces an early and selective enhancement of complex I‐dependent respiration in the skeletal muscle of heart failure patients. This is despite no change in mitochondrial abundance as reflected by unaltered complex I‐V protein levels, and despite no change in skeletal muscle fibre type composition and myoglobin levels (*Graphical Abstract*). Taken together, these data support the notion that IV iron repletion enhances intrinsic skeletal muscle mitochondrial function in heart failure, and that the complex I‐dependent pathway is a site where early enhancements may occur.

Dynamic ^31^P MRS is the only methodology that has so far been used to probe the impact of iron repletion on human skeletal muscle energetics. In FERRIC‐HF II, patients assigned to FDI (*n* = 21) had faster PCr recovery halftimes on ^31^P MRS at 2 weeks compared to those given placebo (*n* = 19): the treatment effect was 8 s.^3^ Subsequently, Frise *et al*.^4^ randomized an iron‐deficient subgroup of healthy individuals to ferric carboxymaltose (*n* = 7) or placebo (*n* = 6). At the lowest exercise workload (3 W), which is optimal for assessing oxidative phosphorylation and broadly similar to the workload applied in FERRIC‐HF II, PCr recovery after 1 week was also faster in the IV iron group, with a similar 8 s treatment effect on the recovery halftime (calculated by multiplying the reported recovery time‐constants [τ] by 0.693). While this was not statistically significant, likely due to the small sample size, the consistency in the direction and magnitude of the treatment effect in both trials supports an energetic basis for some of the benefits of IV iron. However, because ^31^P MRS assesses mitochondrial capacity to generate ATP after muscle contraction *in vivo*, and integrates not only aspects of mitochondrial content and function *per se* but also extra‐mitochondrial factors (e.g. skeletal muscle perfusion and capillary‐myocyte oxygen diffusion), additional methods are needed to resolve the energetic effects of IV iron with greater granularity.

Respirometry is the gold standard means of directly measuring specific ETC complex activities in whole tissue and careful appraisal of our data suggests that our patients harboured exaggerated bioenergetic deficits at baseline. During respirometry, the decline in chamber oxygen levels after the addition of substrates in the absence of ADP is due to background LEAK respiration which compensates for proton leak and slippage and does not contribute to ATP synthesis.^13^ As the main feedback regulator of oxidative phosphorylation rates *in vivo*, ADP titrated to saturating concentrations then ramps up electron flux via the complex I pathway. The responsiveness of the ETC to respiratory control by ADP is measured by the RCR_ADP_ (ratio of respiration supporting ATP synthesis via complex I to that required to offset the proton leak) which is a marker of mitochondrial efficiency that is independent of mitochondrial content. In our cohort, the baseline RCR_ADP_ (2.98) was ~35% lower than that reported in an average heart failure cohort (4.8),^16^ and ~50% lower than that observed in aged healthy individuals (5.00, 6.13, 6.25).^16^, ^17^, ^18^ The ratio of complex I‐linked respiration to maximal coupled respiration (0.45) was also ~25% lower than that reported in both general heart failure patients (0.60)^16^ and aged healthy controls (0.54, 0.56, 0.65, 0.70).^16^, ^17^, ^18^, ^19^ Thus, at baseline, skeletal muscle mitochondria in our cohort appeared to be inefficient and dysfunctional with a lower proportion of electron flow through complex I, i.e. a presumed lower contribution of complex I‐dependent respiration to ATP generation. This is likely due to iron deficiency which, when mild, has been linked to selective reductions in complex I‐dependent respiration in murine cardiac and skeletal muscle.^20^, ^21^

Iron repletion with FDI only augmented complex I and complex I_NET_ activity at 2 weeks implying an early and selective enhancement of complex I‐linked respiration. In the *in‐vivo* setting, this could have occurred at any site along the complex I pathway. First, FDI might have improved energetic cascades upstream of complex I (e.g. glycolysis, Krebs cycle, FA β‐oxidation) leading to a greater delivery of electrons by nicotinamide adenine dinucleotide to complex I. However, as most of these pathways also supply electrons to flavin adenine dinucleotide in complex II, it seems unlikely that FDI would primarily act at these sites and improve complex I‐ but not complex II‐dependent respiration. Second, FDI might have improved components of oxidative phosphorylation downstream of complex I (e.g. coenzyme Q, complex III, complex IV, and phosphorylating system). However, this is unlikely as complex II‐linked respiration also depends on these constituents and did not increase. Thirdly, FDI might have improved skeletal muscle oxygenation by upregulating myoglobin, or increased mitochondrial numbers by inducing a greater proportion of oxidative skeletal muscle fibres that have a higher mitochondrial content. However, the lack of change in the level of myoglobin, oxidative fibres, and complex I‐V proteins (which reflect mitochondrial abundance) with FDI argues against both suggestions. By elimination, therefore, the likeliest explanation is that FDI might have mediated its main effect via complex I.

Iron repletion could have modulated complex I protein activity despite not altering complex I protein content. This is because complex I activity is highly vulnerable to the microenvironment. This vulnerability is conferred by the fact that complex I is the largest of the ETC complexes and has a high number of accessory subunits and Fe‐S clusters. Specifically, complex I has the highest amount of [4Fe‐4S] clusters that are more susceptible to oxidative, nitrosative and inflammatory damage due to their higher redox potential and greater proximity to reactive species.^7^, ^11^, ^12^, ^22^ Additionally, they are more abundant and play more crucial roles in electron transfer than [2Fe‐2S] clusters or haem, making them better targets for regulation and disruption. Attrition of Fe‐S clusters has been reported in mice rendered anaemic from iron deficiency.^23^ Thus, FDI might have augmented complex I activity by replenishing damaged Fe‐S clusters in complex I. Alternatively, FDI might have replenished Fe‐S clusters in the accessory subunits of complex I or induced a conformational shift in complex I from an inactive to an active form.^12^ While our substudy cannot answer these deeper mechanistic questions, the findings are of clinical relevance.

Our study has potential clinical ramifications. First, it should further reassure clinicians that the benefits of IV iron in heart failure, such as improved symptoms and exercise capacity, have a sound mechanistic underpinning which should aid clinical uptake of IV iron repletion. Second, our data imply that the improvements in post‐exercise PCr recovery observed with FDI in FERRIC‐HF II might, in part, be explained by improvements in ETC activity which could be further targeted to maximise patient outcomes. Additional strategies to enhance ETC activity could include the use of coenzyme Q supplements or exercise training.^24^, ^25^ Although only complex I‐linked respiration was enhanced early by FDI, this is still clinically meaningful as complex I sets the pace for oxidative phosphorylation and an increase in its protein content and/or activity is one of the mechanisms via which other beneficial therapies in heart failure, such as exercise, work.^25^ Indeed, only complex I protein levels increased from baseline to week 2 in the placebo group, which likely reflects an adaptive mitochondrial response to the repeated exercise testing incorporated into the study protocol. Participants underwent two 6‐min walk tests, two cardiopulmonary exercise tests, and two in‐magnet ^31^P MRS exercise bouts, representing a substantial cumulative skeletal muscle stimulus. Third, our study reinforces the importance of clinicians actively seeking, investigating, and promptly correcting, systemic iron deficiency in this cohort. Fourth, it suggests that the presence of energetic deficits could be used to identify heart failure patients with iron deficiency who may benefit the most from IV iron. In the absence of dedicated biomarkers (e.g. ^31^P MRS, skeletal muscle ETC assays), such deficits may be approximated clinically through functional assessments. A reduced peak oxygen consumption on cardiopulmonary exercise testing offers the most specific correlation with mitochondrial dysfunction, while a diminished 6‐min walking distance is less specific but provides a pragmatic alternative in resource‐limited settings.^26^, ^27^ Additionally, delayed oxygen uptake during submaximal exertion (on‐kinetics) and slower recovery following exercise (off‐kinetics) are considered dynamic, non‐invasive markers of systemic mitochondrial impairment.^28^, ^29^

Our study's strength is the use of the gold standard respirometry method to assess intrinsic skeletal muscle mitochondrial function within the rigour of a randomized, double‐blind, placebo‐controlled trial. The study also had limitations. First, we chose a short trial duration to minimize attribution of results to skeletal muscle exercise adaptation, so the effect of FDI on ETC activity might have been greater with a longer trial. Second, skeletal muscle iron levels were not quantified due to the larger tissue sample requirements, the complexity of the measurements, and the poor correlation between tissue and systemic iron levels. Third, no adjustments were made for multiple statistical testing as this was a substudy within a clinical trial intended to produce hypothesis‐generating findings rather than definitive conclusions. Fourth, the effect of FDI on total complex I activity was sensitive to imputation likely due to sample size attrition from some of the patients refusing a second skeletal muscle biopsy. However, FDI improved complex I_NET_ respiration with and without imputation. This is crucial as complex I_NET_ reflects complex I‐linked respiration that is truly available for ATP generation.^15^

In conclusion, this is the first study to show that iron repletion with FDI induces an early and selective enhancement of complex I‐linked respiration in the skeletal muscle of heart failure patients. This is despite no change in mitochondrial abundance as reflected by unaltered complex I‐V protein levels, and despite no change in skeletal muscle myoglobin content and fibre type composition. Taken together, these data support the notion that FDI enhances intrinsic skeletal muscle mitochondrial function and that the complex I‐dependent pathway is a site where early enhancements may occur.

## Supporting information


**Appendix S1.** Supporting Information.
