# Supplementary figures and images for: Ferric derisomaltose augments intrinsic skeletal muscle electron transport chain activity in heart failure: A FERRIC‐HF II molecular substudy
Source: Eur J Heart Fail. 2025 Sep 8;27(11):2343–51. doi: 10.1002/ejhf.70028 (PMC12765364; doi:10.1002/ejhf.70028)

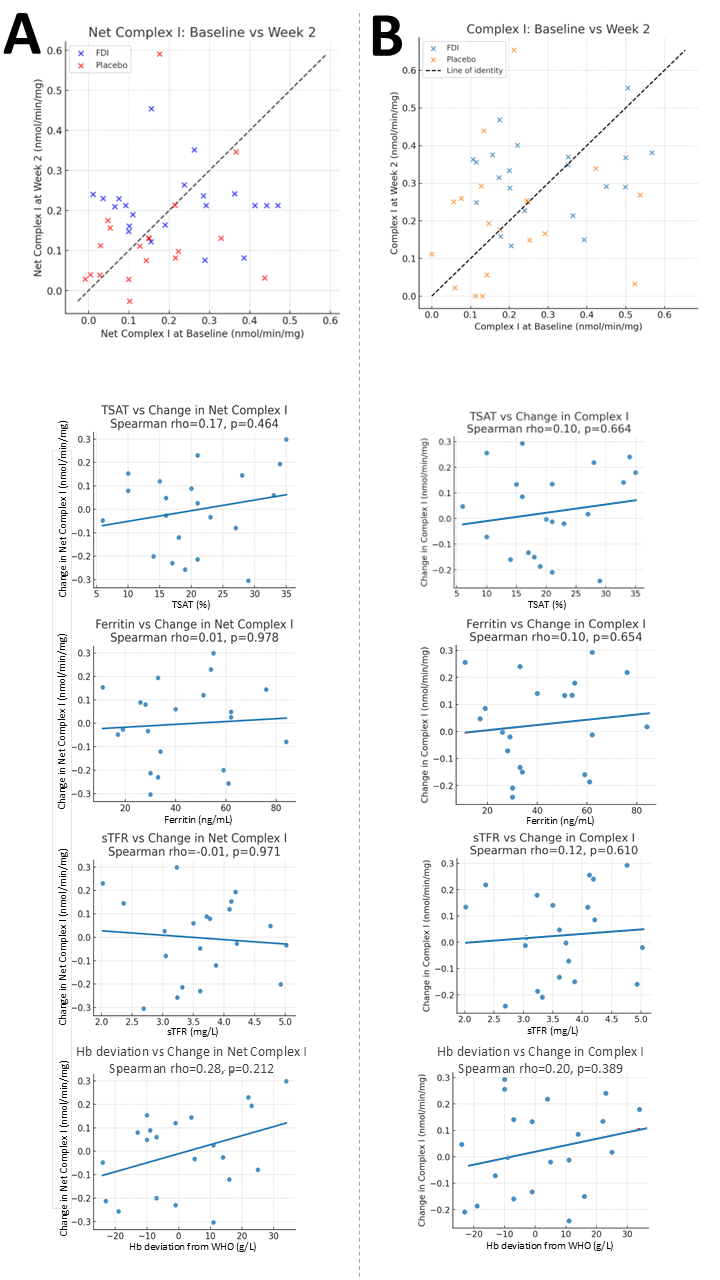

Supplement: Supplementary file 1 — Appendix S1. Supporting Information. [file EJHF-27-2343-s001.tif]
